# Stanniocalcin 1 promotes lung metastasis of breast cancer by enhancing EGFR–ERK–S100A4 signaling

**DOI:** 10.1038/s41419-023-05911-z

**Published:** 2023-07-04

**Authors:** Anfei Liu, Yunting Li, Sitong Lu, Chunqing Cai, Fei Zou, Xiaojing Meng

**Affiliations:** Department of Occupational Health and Occupational Medicine, Guangdong Provincial Key Laboratory of Tropical Disease Research, School of Public Health, Southern Medical University, Guangzhou, Guangdong 510515 China

**Keywords:** Breast cancer, Metastasis

## Abstract

Lung metastasis is the leading cause of breast cancer**-**related death. The tumor microenvironment contributes to the metastatic colonization of tumor cells in the lungs. Tumor secretory factors are important mediators for the adaptation of cancer cells to foreign microenvironments. Here, we report that tumor-secreted stanniocalcin 1 (STC1) promotes the pulmonary metastasis of breast cancer by enhancing the invasiveness of tumor cells and promoting angiogenesis and lung fibroblast activation in the metastatic microenvironment. The results show that STC1 modifies the metastatic microenvironment through its autocrine action on breast cancer cells. Specifically, STC1 upregulates the expression of S100 calcium**-**binding protein A4 (S100A4) by facilitating the phosphorylation of EGFR and ERK signaling in breast cancer cells. S100A4 mediates the effect of STC1 on angiogenesis and lung fibroblasts. Importantly, S100A4 knockdown diminishes STC1**-**induced lung metastasis of breast cancer. Moreover, activated JNK signaling upregulates STC1 expression in breast cancer cells with lung-tropism. Overall, our findings reveal that STC1 plays important role in breast cancer lung metastasis.

## Introduction

Breast cancer has become the most prevalent cancer in the world and the leading cause of cancer death in women [1]. The overwhelming majority of deaths associated with breast cancer are caused by metastases to distant organs, such as the bones and lungs [2]. Triple-negative breast cancer (TNBC) is typically more aggressive and has a greater tendency to metastasize to the lungs [3]. The tumor microenvironment, consists of a variety of cellular and acellular components, plays critical roles in tumor metastasis [4, 5]. Tumor-secreted proteins are the main mediators for the adaptation and modification of cancer cells to external microenvironments [6–8]. Therefore, the discovery of tumor-secreted proteins that can affect or determine tumor metastasis may provide potential prognostic markers and therapeutic targets for tumor intervention.

One of the hallmarks of tumor growth and metastasis is angiogenesis [9]. Similar to normal organs, tumors are required to establish a blood supply to meet their nutritional and oxygen demands, which is achieved primarily through angiogenesis [10]. Tumor angiogenesis is a multidimensional and complex process jointly regulated by cancer cells, numerous tumor-associated stromal cells, and a variety of their bioactive products, including cytokines and growth factors, extracellular matrix, and secreted microvesicles [11]. Since angiogenesis is crucial for tumor growth and metastasis, antiangiogenic therapy has become one of the important therapeutic strategies for tumor treatment [12, 13]. As a pivotal component of the tumor microenvironment, cancer-associated fibroblasts (CAFs) modulate the biological properties of tumor cells and other stromal cells through intercellular contact, release multiple regulatory factors, synthesize and reshape the extracellular matrix, and thus influence the development of cancer [14–16]. Given the abundance of pulmonary vessels and lung fibroblasts in the lung microenvironment, they have an important potential to engage in the development of tumors that metastasize to the lungs [17–20]. However, the specific mechanism of how cancer cells activate them remains to be elucidated.

Stanniocalcin 1 (STC1), a glycoprotein originally isolated from the corpuscles of Stannius in bony fish, is thought to be an endocrine hormone regulating serum calcium and phosphate homeostasis in fish [21]. The human *STC1* gene is broadly expressed in a variety of tissues and performs important functions in multiple physiological and pathological processes, including pregnancy [22], oxidative stress [23], cerebral ischemia [24], and ischemia/reperfusion renal injury [25]. Previous studies have emphasized STC1 as an oncogene in the development of tumors [26]. STC1 has been considered to be involved in tumor proliferation [27], survival [28], metastasis [29, 30], stem cell properties [31, 32], and immune escape [33]. Despite our knowledge that a variety of cells can secrete STC1, the functions of secretory STC1 in cancer remains poorly understood. In this study, we present that tumor cell-derived STC1 contributes to pulmonary metastasis of breast cancer via regulation of angiogenesis and lung fibroblast inflammation in the metastatic microenvironment.

## Materials and methods

### Cell lines and cell culture

Human TNBC cell line MDA-MB-231 was acquired from the Cell Bank/Stem Cell Bank (SIBCB, CAS). LM2-4175 was a gift from the Shanghai Institute of Nutrition and Health (SINH) of the Chinese Academy of Sciences. Human lung fibroblasts MRC5 and human umbilical vein endothelial cells (HUVECs) were acquired from ATCC (Manassas, VA, USA). The cell lines’ authenticity was confirmed by short tandem repeat (STR) profiling. All cells were cultured in high-glucose Dulbecco’s modified Eagle’s medium (DMEM, Gibco, MA, USA) containing 10% fetal bovine serum at 37 °C in a humidified atmosphere of 5% CO_2_.

### Lentivirus stable cell line generation

Construction of gene-stable knockdown and overexpression cell lines with commercially packaged lentiviral vectors (HanBio, Shanghai, China). Lentiviral infection and stable expression clone selection were performed following the manufacturer’s instructions. In brief, breast cancer cells were inoculated in 12-well plates 24 h before treatment, then incubated with polybrene (5 μg/mL) at 37 °C for 30 min and infected with lentivirus for 24 h. Then, replacement of medium was loaded with lentivirus with fresh medium. After 72 h, 1 μg/mL of puromycin was supplemented to the medium for the selection of stable expression clones. The selection process should continue for at least two weeks, during which the cells were cultured normally. The stably expressed cell lines were verified by real-time quantitative PCR (qRT-PCR) and western blot.

### RNA extraction and real-time quantitative PCR

We extracted total RNA from cultured cells with AG RNAex Pro Reagent (AGbio, Hunan, China), and performed cDNA synthesis with 1 μg of total RNA with Evo M-MLV RT Kit (AGbio) based on the manufacturer’s instructions. We performed qRT-PCR to detect relevant mRNA expression with an SYBR® Green Premix Pro Taq HS qPCR Kit (AGbio) and a CFX Connect 96 Real-Time PCR System (Bio-Rad, Singapore). The expression level of mRNA relative to GAPDH was assessed by the 2^−ΔΔCt^ method. The qRT-PCR primer sequences are listed in Supplementary Table 1.

### Western blot

Cell pellets were lysed in the RIPA lysis buffer (KeyGEN, Nanjing, China) that contains 1% protease and phosphatase inhibitor cocktail (KeyGEN, Nanjing, China) for 30 min on ice. Then, lysates were centrifuged at 12,000×*g* for 15 min at 4 °C. The supernatants of the lysates were harvested for concentration determination and denatured at 100 °C. Denatured protein extracts measuring 40 μg were fractionated by 8–12% sodium dodecyl sulfate-polyacrylamide gel electrophoresis (SDS-PAGE) and transferred to polyvinylidene fluoride (PVDF) membranes. Following blocking in TBST containing 5% bovine serum albumin for 1 h at room temperature, the membranes were incubated with primary antibodies (see Supplementary Table 2) diluted in Universal Antibody Diluent (NCM Biotech, Suzhou, China) overnight at 4 °C. On the second day, IRDye goat anti-mouse or rabbit secondary antibodies (Gene Company, Shanghai, China) were used to incubate the membranes at room temperature for 1 h. An LI-COR Odyssey infrared imaging system (LI-COR Biosciences, USA) was used for the visualization of protein blots. The Gray analysis of the target protein bands was performed with ImageJ and all protein expression levels were normalized to the GAPDH protein level of each sample. Comparisons were then made with the corresponding controls.

### Cell Counting Kit-8 assay

An appropriate number of cells were inoculated in 96-well cell culture plates for 24 h before intervention. The Cell Counting Kit-8 (CCK-8) assay (Dojindo Laboratories, Shanghai, China) was used to evaluate cell viability or proliferation based on the manufacturer’s instructions.

### Transwell migration and invasion assays

The transwell chamber (#3422, Corning Incorporated, Corning, NY, USA) was employed for the transwell invasion assay. Matrigel (#356234, Corning Incorporated, Corning, NY, USA) was thawed on ice overnight before use. The upper chamber surface of the transwell inserts was coated with a diluted solution of Matrigel (1:8) and dried in the incubator. This was followed by adding 50 μL of serum-free medium to each well and hydrating the basement membrane at 37 °C for 30 min. Serum deprivation starving cells for 12–24 h. Harvest the cells into a serum-free medium and adjust the cell density to 1 × 10^5^ cells/mL. The transwell insert was filled with 200 μL of cell suspension and the bottom chamber was filled with 600 μL of complete or conditioned medium as a chemoattractant. After 36 h culture, A swab was used to clean the cells left in the upper chamber of the transwell insert, and the invaded cells were fixed with 4% paraformaldehyde and stained with 0.1% crystal violet. Under an inverted microscope, stained cells were pictured and counted in six randomly selected fields.

The transwell migration assay was carried out in a similar manner as the transwell invasion assay, except that the transwell chamber in the cell migration assay did not need to be coated with Matrigel before the assay.

### Tube formation assay

Matrigel (#356234, Corning) was thawed on ice overnight and added to μ-slide (#81506; Ibidi, Martinsried, Germany) at 10 μL/well, then solidified for 30 min at 37 °C incubator. HUVECs were harvested and suspended in DMEM containing 10% fetal bovine serum at a density of 1 × 10^5^ cells/mL. Subsequently, HUVECs suspension (50 μL) was added to each well and μ-slide was incubated at 37 °C for 4 h in a humidified atmosphere of 5% CO_2_. Then, the μ-slide was visualized under an inverted microscope and images were acquired. AngioTool was used to analyze the tube formation capacity of the closed networks of vessel-like tubes [34].

### Tumorigenesis and lung metastasis assays in nude mice

Four-week-old female BALB/c nude mice were purchased from the Laboratory Animal Center of Southern Medical University (Guangzhou, China). The animals were cared for in accordance with the legal mandates and national guidelines, and the protocols were approved by the Animal Ethics Committee of Southern Medical University (Authorization No. 2020064).

For the tumorigenesis assay, cells were collected by trypsin method, washed with PBS, and counted. Then, the cells were resuspended in PBS, the cell density was adjusted to 10^7^ cells/mL, and 0.1 mL of cell suspension was inoculated into a mouse mammary gland fat pad. Tumor outgrowth was monitored by recording the tumor length (L) and width (W) every 3 days for 4 weeks. Tumor volume was calculated as LW^2^/2. The tumors were weighed and photographed at the end of the experiment. For lung metastasis assay, viable cells were washed and collected in PBS and subsequently, 10^6^ cells in 100 μl PBS were injected into the tail vein. Endpoint measurements were performed at 6 weeks after injection, unless there was a significant morbidity that requires early euthanasia of the mice. The lungs were harvested, perfused with normal saline, and fixed with Bouin’s solution. Pulmonary tumor nodules were counted under a stereoscopic microscope.

### Immunohistochemical staining

Paraffin-embedded breast cancer and lung tissue specimens were sectioned to 4-μm thickness. The tissue slides were baked at 60 °C for 2 h. Following deparaffinization and hydration, the tissue slides were boiled in citrate buffer at 100 °C for 30 min. After blocking endogenous peroxidase by treatment with 3% H_2_O_2_, the tissue slides were incubated with primary antibodies (see Supplementary Table 2) at 4 °C overnight. On the second day, the HRP-conjugated goat anti-mouse or rabbit secondary antibody were used to incubate the tissue slides at room temperature away from light for 1 h. Slices were stained with DAB, counterstained with hematoxylin, and then sealed with Ramsan gum.

### mRNA sequencing

Following the manufacturer’s instructions, we extracted total RNA with a TRIzol reagent (Takara, Japan). We used the RNA 6000 Nano Assay Kit and the 2100 Bioanalyzer System (Agilent Technologies, CA, USA) to evaluate the concentration, purity, and integrity of RNA. Novogene Co., Ltd. (Beijing, China) conducted the library preparation and transcriptome sequencing. Genes with adjusted *p* value <0.05 and |log2 (Fold Change)| >0 were identified as differentially expressed.

### Chromatin immunoprecipitation–qPCR

We used the SimpleChIP Enzymatic Chromatin IP Kit (magnetic beads) (Cell Signaling, #9003) to perform chromatin immunoprecipitation assays following the manufacturer’s instructions. The antibodies used were c-Jun (60A8) Rabbit mAb (Cell Signaling, #9165) and Normal Rabbit IgG (Cell Signaling, #2729). The following qPCR primers were used: Primer1-F: ACCAGTGGCCATTTCTCATC, Primer1-R: CTGAGGTTTGTCCCCAACAT, Primer2-F: GGGTGGCACTCTTTCTTCAA, and Primer2-R: AATGGCCACTGGTGATTCAT.

### Bioinformatics

The STC1 mRNA expression data of breast cancer patients were obtained from the Oncomine database. The Kaplan–Meier Plotter database (https://kmplot.com/analysis/) was accessed to predict the effect of STC1 on the survival and prognosis of breast cancer patients. The association of STC1 (204595_s_at) gene expression levels with the overall survival (OS) and distant metastasis-free survival (DMFS) was automatically analyzed by drawing a Kaplan–Meier plot. The mRNA expression levels of STC1 and survival data of breast cancer patients were from breast cancer datasets accessed from the Gene Expression Omnibus (www.ncbi.nlm.nih.gov/geo/). The GSE2034, GSE5327, and GSE2603 datasets were selected to analyze the association of STC1 (204595_s_at) gene expression levels with lung metastasis-free survival (LMFS).

### Statistical analysis

GraphPad Prism (Version 8.0) was used to perform the data analysis. The data were from at least three independent experiments to ensure reproducibility and presented as mean ± standard deviation. Student’s *t*-test, one-way ANOVA test, or repeated-measures two-way ANOVA test were used for statistical analysis, and *p* values <0.05 were considered statistically significant. Survival analysis was performed using Kaplan–Meier analysis and log-rank test. The correlation between different data were assessed by the Pearson correlation coefficient. All statistical tests were two-sided.

## Results

### STC1 is associated with breast cancer lung metastasis

**Fig. 1:**
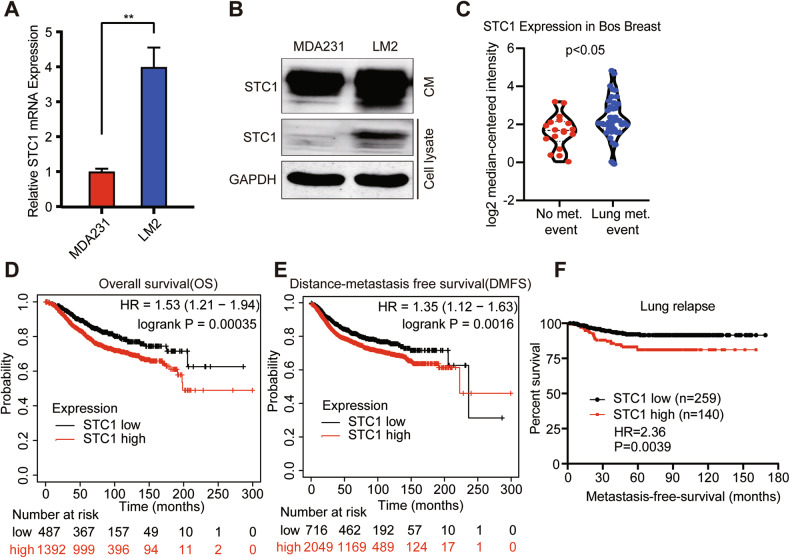
STC1 is associated with breast cancer lung metastasis. **A** Relative mRNA expression levels of STC1 in breast cancer cell lines MDA-MB-231 and LM2 were detected by RT-qPCR. **B** Expression level of STC1 protein in breast cancer cell lines MDA-MB-231 and LM2 were determined by western blot. **C** The mRNA expression data of STC1 in breast cancer patients was obtained from the oncomine database. **D**, **E** OS (**D**) and DMFS (**E**) of breast cancer patients according to the mRNA expression level of STC1 in the KM Plotter database. **F** LMFS of breast cancer patients according to the mRNA expression level of STC1 in the GEO datasets. The *p* values were determined by unpaired *t*-test (**A**), Mann–Whitney test (**C**), or log-rank test (**D**–**F**). CM conditioned medium, STC1 stanniocalcin 1, KM Kaplan–Meier, GEO Gene Expression Omnibus. **p < 0.01.

To investigate the contribution of STC1 to pulmonary metastasis of breast cancer, we checked the STC1 expression in breast cancer cell lines with different pulmonary metastatic potential, including TNBC cell line MDA-MB-231 and its pulmonary tropism derivative LM2-4175 (LM2) [35]. We observed that STC1 mRNA expression levels in LM2 cells was markedly higher than that in MDA-MB-231 cells (Fig. 1A). The intracellular and extracellular STC1 protein levels in LM2 cells were higher than those in MDA-MB-231 cells (Fig. 1B).

To further verify the clinical significance of STC1 expression, we mined the expression of STC1 in breast cancer patients in the Oncomine database. It was found that the STC1 was significantly upregulated in breast cancer patients with lung metastasis as compared with those without distant metastasis (Fig. 1C). Moreover, we evaluated the association of STC1 expression levels with the survival of breast cancer patients in the Kaplan–Meier Plotter database. The results indicated that the high expression of STC1 in tumor samples of breast cancer patients was linked to shortened OS and DMFS of breast cancer patients (Fig. 1D, E). Furthermore, we queried the association between STC1 expression and lung metastasis in a combined breast cancer microarray data set (GSE2034, GSE2604, and GSE5327) and found that the high STC1 expression was significantly associated with lower LMFS in breast cancer patients (Fig. 1F). These data showed a positive association for STC1 and lung metastasis of breast cancer.

### STC1 promotes lung metastasis of breast cancer

**Fig. 2:**
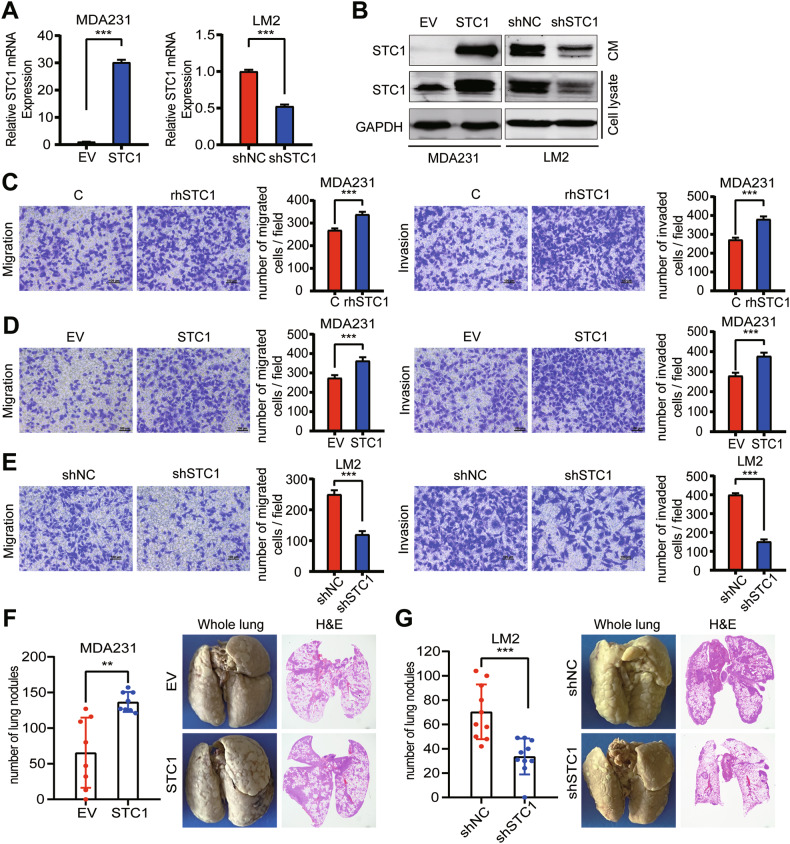
STC1 promotes lung metastasis of breast cancer. **A**, **B** Overexpression and knockdown of STC1 in human breast cancer cell lines MDA-MB-231 and LM2 were verified by RT-qPCR (**A**) and Western blot (**B**). **C** Transwell migration and Matrigel invasion of rhSTC1-treated MDA-MB-231 cell. **D** Transwell migration and Matrigel invasion of STC1-overexpressed MDA-MB-231. **E** Transwell migration and Matrigel invasion of LM2 after STC1 knockdown. **F** Lung metastasis nodule quantification and representative images after tail vein injection of STC1-overexpressed MDA-MB-231 cells (*n* = 8 mice per group). **G** Lung metastasis nodule quantification and representative images after tail vein injection of STC1 knocked-down LM2 cells (*n* = 10 mice per group). The *p* values were determined by unpaired *t*-test. Scale bars, 100 μm. STC1 stanniocalcin 1, rhSTC1 recombinant human STC1, H&E hematoxylin and eosin staining. **p < 0.01; ***p < 0.001.

First, we checked the roles of STC1 on tumor cell–innate malignancy and noticed that cell proliferation was not affected after recombinant human STC1 (rhSTC1) treatment (Fig. S1A). Neither STC1 overexpression in MDA-MB-231 nor STC1 knockdown in LM2 affected cell proliferation (Figs. 2A, B and S1B). However, we found that rhSTC1 treatment and STC1 overexpression enhanced cell migration and invasion and that STC1 knockdown exerted the opposite effect (Fig. 2C–E).

We then examined the regulatory effect of STC1 in pulmonary metastasis. STC1 overexpression significantly aggravated the burden of lung metastasis in mice after intravenous injection of MDA-MB-231 cells (Fig. 2F). Conversely, STC1 knockdown in LM2 reduced lung colonization (Fig. 2G). Moreover, we implanted the MDA-MB-231 cells into the mammary fat pads of BALB/c nude mice and found that STC1 had no impact on the growth of primary breast tumors (Fig. S1C). These findings showed the pro-metastatic activity of STC1 in breast cancer.

### STC1 indirectly promotes angiogenesis in breast cancer lung metastases

**Fig. 3:**
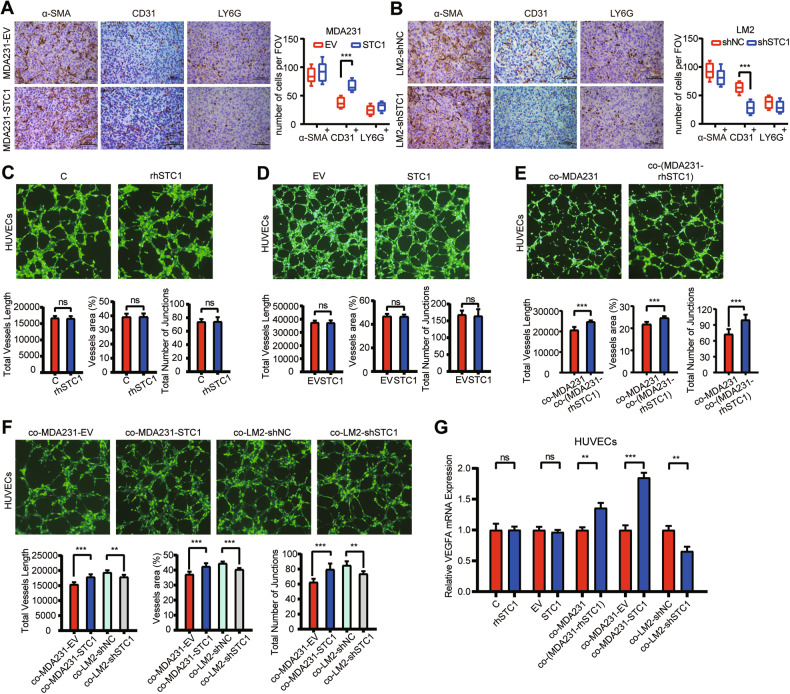
STC1 promotes angiogenesis in breast cancer lung metastases. **A**, **B** IHC analyses of α-SMA^+^, CD31^+^, and LY6G^+^ cells in the lung metastases of breast cancer; *n* = 6 samples from three mice per group. **C** Tube formation of rhSTC1-treated HUVECs. **D** Tube formation of STC1-overexpressing HUVECs. **E** Tube formation of HUVECs co-cultured with rhSTC1-pretreated MDA-MB-231. **F** Tube formation of HUVECs co-cultured with STC1-overexpressing MDA-MB-231 or STC1-downregulated LM2. The tube-forming capacity of HUVECs was evaluated by vessel length, percentage of vessel area, and the number of vascular junction points in (**C**–**F**). **G** Relative mRNA expression levels of VEGFA in HUVECs under corresponding treatment or co-culture conditions. The *p* values were obtained by unpaired *t*-test. Scale bars, 100 μm. STC1 stanniocalcin 1, IHC immunohistochemistry, rhSTC1 recombinant human STC1. ns no significance, *p* > 0.05; **p < 0.01; ***p < 0.001.

As STC1 is a breast cancer cell-derived secreted protein, it is necessary to detect microenvironmental alteration of pulmonary metastases after STC1 manipulation. Multiple stromal components in the tumor microenvironment, especially fibroblasts, endothelial cells, and neutrophils, have been proved to play important roles in cancer metastasis to the lungs [17, 18, 36]. We carried out immunostaining of α-SMA, CD31, and LY6G to characterize the aggregation of fibroblasts, endothelial cells, and neutrophils in lung metastases, respectively. The results indicated that STC1 overexpression augmented, whereas knockdown repressed, the percentages of CD31^+^ vascular endothelial cells but not other stromal elements in lung metastases (Fig. 3A, B). These results implied that STC1 could promote angiogenesis in lung metastases of breast cancer.

We further investigated the effect of STC1 on the tubule formation ability of HUVECs. We found that the parameters characterizing the angiogenic capacity of HUVECs remained unchanged after rhSTC1 treatment (Fig. 3C). STC1 overexpression in HUVECs could not enhance tubule formation (Fig. 3D). However, HUVECs co-cultured with rhSTC1-pretreated MDA-MB-231 cells formed more tubule junctions and longer vessels length and a greater percentage of vessels area (Fig. 3E). Furthermore, the tube formation ability of HUVECs co-cultured with MDA-MB-231-STC1 cells was remarkably stronger than that of HUVECs co-cultured with MDA-MB-231-EV cells, whereas the tube formation ability of HUVECs co-cultured with LM2-shSTC1 cells was significantly weaker than that of HUVECs co-cultured with LM2-shNC cells (Fig. 3F). Moreover, the relative expression levels of VEGFA in HUVEC cells under corresponding treatment or co-culture conditions supported the phenomenon observed in the tube formation assay (Fig. 3G). Taken together, these data showed that STC1 promotes angiogenesis in some indirect way.

### STC1 promotes fibroblast activation in breast cancer lung metastasis

**Fig. 4:**
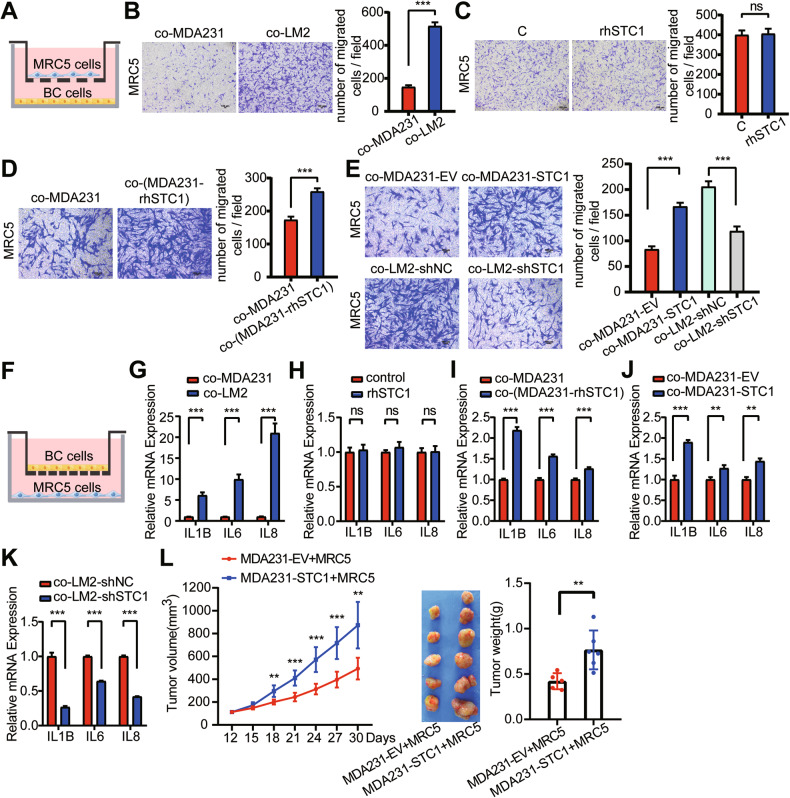
STC1 promotes fibroblast activation in breast cancer lung metastasis. **A** Schematic diagram of transwell migration assay. **B** Transwell migration of MRC5 co-cultured with MDA-MB-231 or LM2. **C** Transwell migration of MRC5 treated with rhSTC1. **D** Transwell migration of MRC5 co-cultured with rhSTC1-pretreated MDA-MB-231. **E** Transwell migration of MRC5 co-cultured with STC1-overexpressing MDA-MB-231 or STC1-downregulated LM2. **F** Schematic diagram of transwell co-culture assay. **G**–**K** RT-qPCR was performed to detect the relative mRNA expressions of inflammatory factors IL1B, IL6, and IL8. The relative mRNA expressions of inflammatory factors IL1B, IL6, and IL8 in MRC5 co-cultured with MDA-MB-231 or LM2 (**G**). The relative mRNA levels of inflammatory factors IL1B, IL6, and IL8 in MRC5 treated with rhSTC1 (**H**). The relative mRNA levels of inflammatory factors IL1B, IL6, and IL8 in MRC5 co-cultured with MDA-MB-231 pretreated with rhSTC1 (**I**). The relative mRNAs expression of inflammatory factors IL1B, IL6, IL8 in MRC5 co-cultured with STC1-overexpressing MDA-MB-231 or STC1-downregulated LM2 (**J**, **K**). **L** Volume and weight of tumors formed by subcutaneously injecting a mixture of MRC5 and MDA-MB-231 in nude mice. The *p* values were obtained by unpaired *t*-test or repeated-measures two-way ANOVA test. Scale bars, 100 μm. BC cells breast cancer cells, STC1 stanniocalcin 1, rhSTC1 recombinant human STC1. ns no significance, *p* > 0.05; **p < 0.01; ***p < 0.001.

Although the immunostaining of breast cancer lung metastases showed no change in the number of α-SMA-positive cells after STC1 manipulation (Fig. 3A, B), the immunostaining of α-SMA may not reflect the activation of lung fibroblasts because of the extremely abundant fibroblasts in the lungs. Therefore, we further tested the effect of STC1 on lung fibroblast MRC5 migration by transwell migration assay (Fig. 4A–E). We found that LM2 cells had a much stronger ability to promote lung fibroblast MRC5 migration than MDA-MB-231 cells under transwell co-culture condition (Fig. 4B), but the rhSTC1 treatment did not affect the migration of MRC5 cells (Fig. 4C). We found that MDA-MB-231 cells pretreated with rhSTC1 could attract a higher number of MRC5 cells to migrate through the membrane pores (Fig. 4D). The migrated number of MRC5 cells co-cultured with MDA-MB-231–STC1 cells was prominently larger than that of MRC5 cells co-cultured with MDA-MB-231-EV cells, whereas the migrated number of MRC5 cells co-cultured with LM2-shSTC1 cells was significantly smaller than that of MRC5 cells co-cultured with LM2-shNC cells (Fig. 4E).

In the tumor microenvironment, fibroblasts can be “educated” by tumor cells to turn into tumor-associated fibroblasts that exhibit pro-inflammatory properties [14, 15]. Therefore, we further tested the effect of STC1 on inflammatory changes of lung fibroblasts MRC5 by transwell co-culture assay (Fig. 4F–K). We found that the expressions of pro-inflammatory cytokines IL1B, IL6, and IL8 in MRC5 cells co-cultured with LM2 cells were markedly higher than those in MRC5 cells co-cultured with MDA-MB-231 cells (Fig. 4G). Therefore, can STC1 cause inflammatory changes in lung fibroblasts? We found that the expressions of these pro-inflammatory cytokines in MRC5 cells exhibited no significant changes after rhSTC1 treatment (Fig. 4H). However, MRC5 cells co-cultured with rhSTC1-pretreated MDA-MB-231 cells expressed higher levels of IL1B, IL6, and IL8 (Fig. 4I). Furthermore, the expression levels of pro-inflammatory cytokines IL1B, IL6 and IL8 in MRC5 cells co-cultured with MDA-MB-231-STC1 cells were significantly higher than those in MRC5 cells co-cultured with MDA-MB-231-EV cells (Fig. 4J), whereas the expression levels of these pro-inflammatory cytokines in MRC5 cells co-cultured with LM2-shSTC1 cells were significantly lower than those of MRC5 cells co-cultured with LM2-shNC cells (Fig. 4K). Taken together, these data showed that STC1 promoted fibroblast activation in some indirect way. Further, we subcutaneously inoculated nude mice with a mixture of MRC5 cells and MDA-MB-231 cells and found that STC1 promoted breast cancer growth in the presence of MRC5 cells (Fig. 4L).

### S100A4 is a target of STC1 in breast cancer cells

**Fig. 5:**
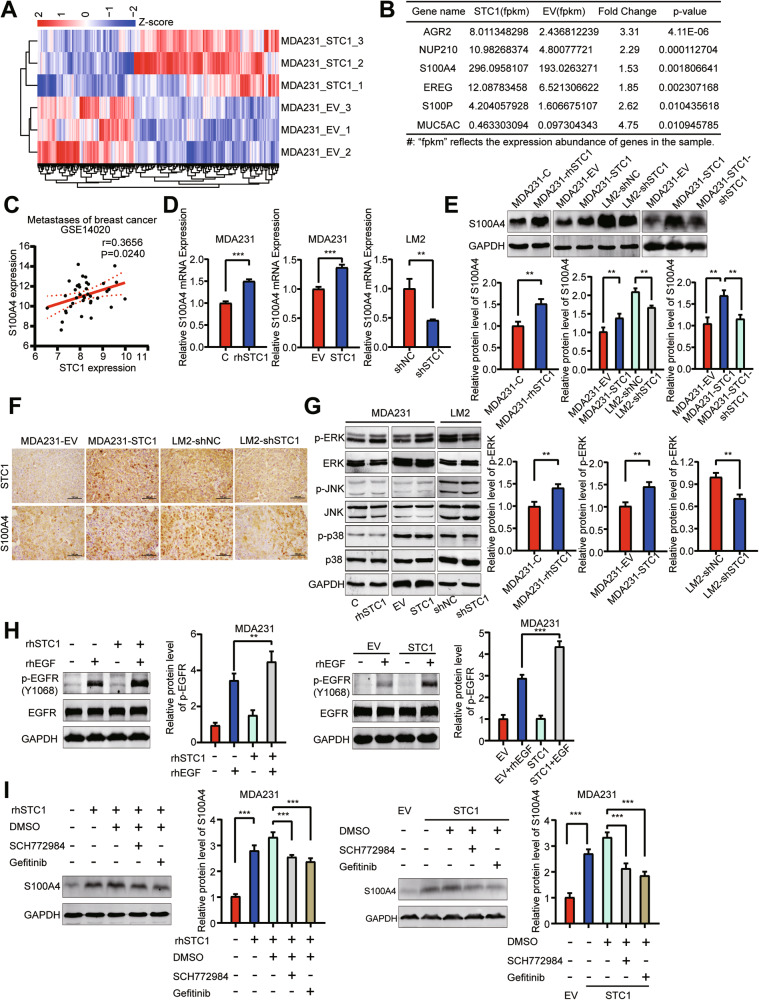
S100A4 is a target of STC1 in breast cancer cells. **A** Heatmap of cluster analysis of differentially expressed genes in transcriptome sequencing. **B** Screening of differentially expressed genes related to lung metastasis in transcriptome sequencing data. **C** Correlation analysis of S100A4 and STC1 mRNA expression in metastatic lesions of breast cancer patients (data from GSE14020). **D** The relative mRNA expression of S100A4 affected by STC1 was assayed by RT-qPCR. **E** The protein level of S100A4 affected by STC1 was assessed by Western blot. **F** IHC staining of STC1 and S100A4 in the lung metastases of breast cancer. **G** Western blot analysis of MAPK signaling pathway in MDA-MB-231 (with rhSTC1 (100 ng/ml) treatment or STC1 overexpression) and LM2 (STC1 knockdown). **H** Western blot analysis and the quantification of p-EGFR of MDA-MB-231 cells with or without 1-h pretreatment with 100 ng/mL of rhSTC1, followed by treatment with rhEGF (100 ng/mL) for an additional 10 min (left). Western blot analysis and the quantification of p-EGFR of EV control or STC1-overexpressing MDA-MB-231 cells cultured for 48 h, followed by rhEGF (100 ng/mL) treatment for another 10 min (right). **I** Western blot analysis and the quantification of S100A4 of MDA-MB-231 cells with or without 1-h pretreatment with 100 ng/mL of rhSTC1, followed by treatment with ERK inhibitor SCH772984 (10 μM) or EGFR inhibitor gefitinib (10 μM) for another 24 h (left). Western blot analysis and the quantification of S100A4 of EV control or STC1-overexpressing MDA-MB-231 cells cultured for 48 h followed by treatment with ERK inhibitor SCH772984 (10 μM) or EGFR inhibitor gefitinib (10 μM) for another 24 h (right). The *p* values were obtained by unpaired *t*-test or one-way ANOVA test with Tukey’s multiple comparisons test or Pearson correlation analysis. Scale bars, 100 μm. STC1 stanniocalcin 1, IHC immunohistochemistry, rhEGF recombinant human EGF. **p < 0.01; ***p < 0.001.

The aforementioned results not only showed that STC1 could enhance the invasiveness of breast cancer cells, but also indicated that breast cancer cells mediated the effect of STC1 on the pulmonary metastatic microenvironment. This indicates that breast cancer cell-derived STC1 mainly acts on tumor cells themselves. Next, we inquired into the potential mechanism of STC1-induced promotion of tumor metastasis. We used transcriptome sequencing to identify potential STC1 targets (Fig. 5A). The results revealed that compared with control MDA-MB-231-EV cells, pro-metastatic genes, such as NUP210, S100A4, S100P, AGR2, MUC5AC, and EREG, were upregulated in MDA-MB-231-STC1 cells. Among these pro-metastatic genes, S100A4 had the highest expression abundance (Fig. 5B). S100A4 is considered to be an important driver gene of tumor metastasis and plays a significant role in the lung metastasis of tumors. It can not only promote the invasiveness of tumor cells themselves, but can also regulate the tumor microenvironment [37, 38].

Transcriptome sequencing results showed that S100A4 may be a downstream target molecule of STC1. We found a positive correlation between the expression of S100A4 and STC1 in metastatic lesions of breast cancer patients (Fig. 5C). We found that rhSTC1 treatment could upregulate the mRNA and protein levels of S100A4 in MDA-MB-231 cells (Fig. 5D, E). STC1 overexpression in MDA-MB-231 promoted the mRNA and protein levels of S100A4, whereas STC1 knockdown in LM2 showed the opposite effect (Fig. 5D, E). When STC1 was knocked down again in MDA-MB-231–STC1 cells, the protein expression of S100A4 was also reverted (Fig. 5E). Immunostaining of lung metastases tissue confirmed the phenomena observed in vitro (Fig. 5F).

We further examined the mechanism by which STC1 upregulates S100A4. Previous study has indicated that the JNK signaling pathway can be activated by STC1 in breast cancer cells [39]. We found that rhSTC1 treatment and STC1 overexpression could promote the activation of the MAPK signaling pathway in MDA-MB-231 cells, mainly manifested as the upregulation of the phosphorylated level of ERK protein, whereas the phosphorylation of JNK and p38 protein was not significantly changed (Fig. 5G). These data indicated that STC1 could promote the activation of the ERK signaling pathway in breast cancer cells. After treatment with the ERK signaling inhibitor SCH772984, the protein level of S100A4 upregulated by rhSTC1 was significantly reversed in MDA-MB-231 cells, and a similar result was also observed in MDA-MB-231–STC1 cells (Fig. 5I). This indicated that the STC1-activated ERK signaling upregulates the expression of S100A4. It is known that the ERK signaling pathway can be activated by phosphorylated EGFR [40]. We found that rhSTC1 treatment could not increase the phosphorylation level of EGFR alone, but rhSTC1 treatment enhanced the ability of recombinant human EGF (rhEGF) to phosphorylate EGFR in MDA-MB-231 cells (Fig. 5H). Similarly, after treatment with rhEGF, the level of p-EGFR in MDA-MB-231–STC1 cells was higher than that in MDA-MB-231-EV cells (Fig. 5H). These data indicated that STC1 can facilitate the phosphorylation of EGFR by EGF. Furthermore, after treatment with the EGFR signaling inhibitor gefitinib, the protein level of S100A4 upregulated by rhSTC1 was significantly reversed in MDA-MB-231 cells, and a similar result was observed in MDA-MB-231–STC1 cells (Fig. 5I). These results showed that STC1 promotes EGFR phosphorylation and its downstream ERK signaling and then upregulates S100A4 in lung metastatic breast cancer cells.

### S100A4 mediates the functions of STC1 in breast cancer lung metastasis

**Fig. 6:**
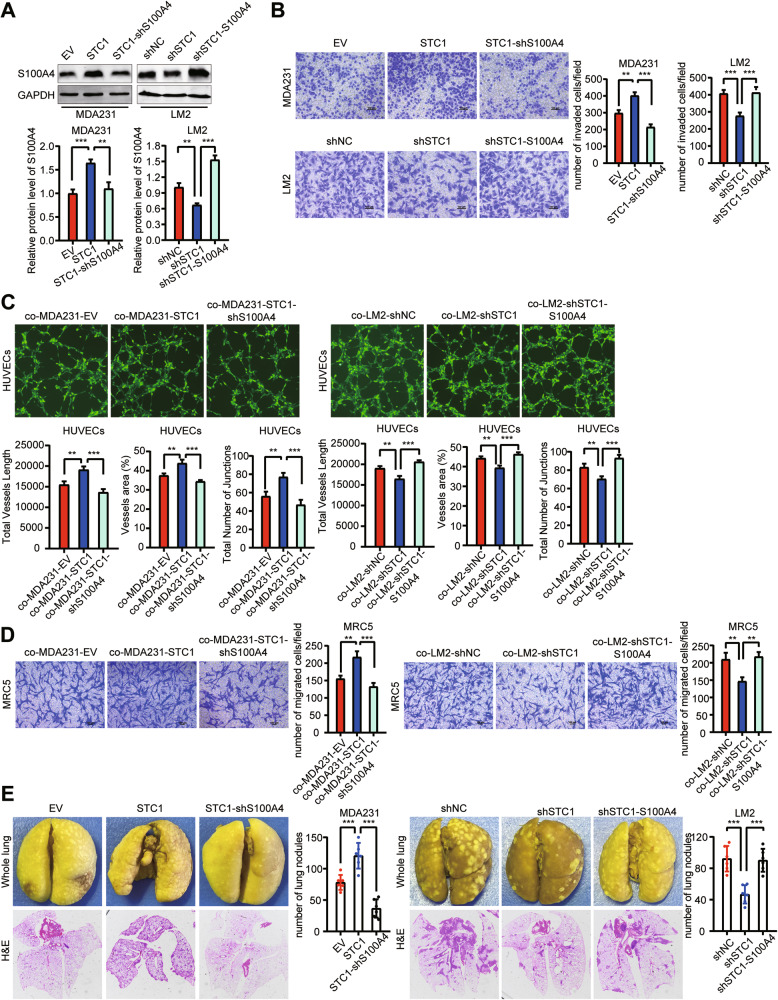
S100A4 mediates the functions of STC1 in breast cancer lung metastasis. **A** Validation of S100A4 knockdown and overexpression in human breast cancer cell lines, MDA-MB-231-STC1 and LM2-shSTC1, by Western blot analyses. **B** Transwell invasion of MDA-MB-231(EV, STC1, and STC1-shS100A4) and LM2 (shNC, shSTC1, and shSTC1-S100A4). **C** Tube formation of HUVECs co-cultured with MDA-MB-231 (EV, STC1, and STC1-shS100A4) or LM2 (shNC, shSTC1, and shSTC1-S100A4). **D** Transwell migration of MRC5 co-cultured with MDA-MB-231 (EV, STC1, and STC1-shS100A4) or LM2 (shNC, shSTC1, and shSTC1-S100A4). **E** Lung metastasis nodule quantification and representative images after tail vein injection of MDA-MB-231 (EV, STC1, and STC1-shS100A4) or LM2 (shNC, shSTC1, and shSTC1-S100A4); *n* = 6 mice per group. The *p* values were obtained by one-way ANOVA test with Tukey’s multiple comparisons test. Scale bars, 100 μm. STC1 stanniocalcin 1. **p < 0.01; ***p < 0.001.

S100A4 is an important driver of tumor lung metastasis [37, 38]. By overexpression and knockdown of S100A4, we demonstrated that S100A4 can promote breast cancer cell invasion, enhance vascular endothelial cell tube formation, and promote fibroblast activation (Fig. S2). We further determined whether S100A4 is involved in mediating the functions of STC1 in promoting the aggression of breast cancer cells, the tubule formation of HUVECs, and the chemotactic migration of lung fibroblasts. The expression of S100A4 was knocked down in MDA-MB-231–STC1 cells and overexpressed in LM2-shSTC1 cells (Fig. 6A). We noticed that the knockdown of S100A4 restored the promotional action of STC1-overexpressing breast cancer cells on these functions, and overexpression of S100A4 rescued the inhibitory effect of STC1 knockdown breast cancer cells on these functions (Fig. 6B–D). Moreover, we found that the knockdown of S100A4 reduced the enhanced lung metastasis ability of MDA-MB-231-STC1 cells, and that the overexpression of S100A4 rescued the impaired lung metastasis ability of LM2-shSTC1 cells (Fig. 6E). These results showed that S100A4 mediates the functions of STC1 in lung metastasis of breast cancer.

### JNK signaling pathway activates the expression of STC1

**Fig. 7:**
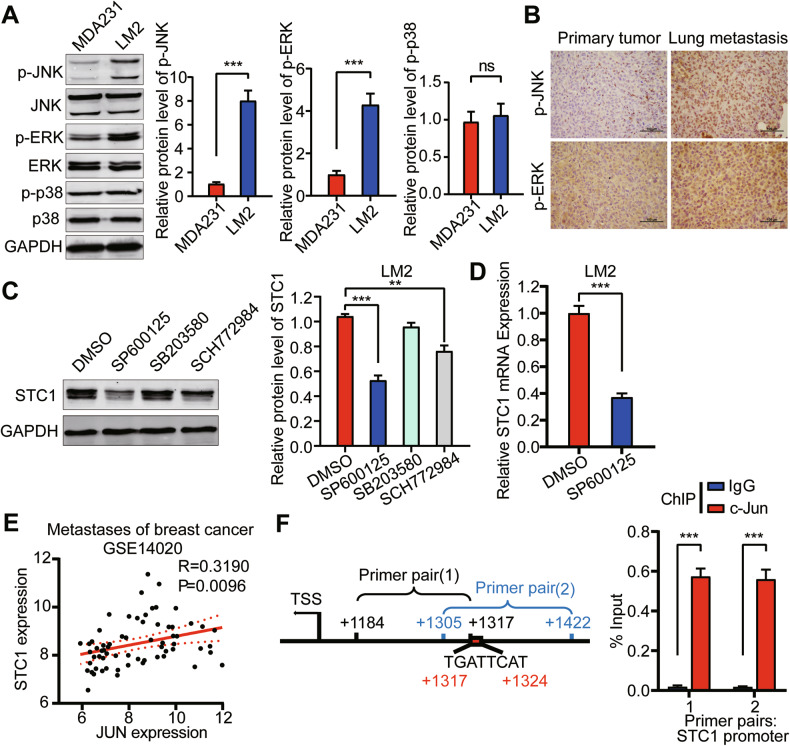
JNK signaling pathway activates the expression of STC1. **A** Western blot analysis of MAPK signaling pathway in MDA-MB-231 and LM2. **B** IHC staining of p-ERK and p-JNK in primary tumors and lung metastases formed by MDA-MB-231 cells. **C** Western blot analysis of STC1 in LM2 treated with MAPK signaling pathway inhibitor (10 μM) for 24 h. **D** RT-qPCR was performed to detect the relative expression of STC1 mRNA in LM2 treated with JNK signaling pathway inhibitor SP600125 (10 μM). **E** Correlation analysis of STC1 and JUN mRNA expression in metastatic lesions of breast cancer patients (data from GSE14020). **F** Schematic diagram of STC1 promoter displaying locations of primer pairs, covering c-Jun binding sites, which were used for qPCR analysis following chromatin immunoprecipitation with an antibody against c-Jun (left). The qPCR analysis of STC1 promoter fragments pulled down by c-Jun and IgG antibodies in chromatin immunoprecipitation (right). The *p* values were obtained by unpaired *t*-test or one-way ANOVA test with Dunnett’s multiple comparisons test or Pearson correlation analysis. Scale bars, 100 μm. STC1 stanniocalcin 1, IHC immunohistochemistry, ChIP chromatin immunoprecipitation. ns no significance, *p* > 0.05; **p < 0.01; ***p < 0.001.

The lung is a relatively high-oxygen environment and faces more external stimuli [41]. To survive and develop continuously in the lungs, tumor cells must adapt to this environmental pressure. As is well known, the MAPK signaling pathway is susceptible to activation by extracellular stimuli [42]. We found that compared with MDA-MB-231 cells, the MAPK signaling pathway was activated in lung metastatic breast cancer LM2 cells, which manifested as increased phosphorylation of ERK and JNK protein (Fig. 7A). Immunostaining indicated that the levels of ERK and JNK phosphorylation in lung metastases formed by MDA-MB-231 cells were higher than those in carcinoma in situ formed by MDA-MB-231 cells (Fig. 7B). This demonstrated that the MAPK signaling pathway was activated in lung metastatic breast cancer cells. STC1 is known to be a cellular stress response factor [21]. Therefore, we next investigated whether the activated MAPK signaling pathway contributes to the expression of STC1. After treatment with MAPK signaling pathway inhibitors for 24 h in LM2 cells, it was found that the JNK inhibitor SP600125 could significantly downregulate the protein level of STC1 (Fig. 7C). Meanwhile, RT-qPCR results showed that the mRNA level of STC1 was significantly downregulated (Fig. 7D). This indicated that the transcription of STC1 mRNA could be regulated by the JNK signaling pathway. C-Jun is the most important transcription factor of the JNK signaling pathway. We found a correlation between the expressions of JUN mRNA and STC1 mRNA in the metastases of breast cancer patients, suggesting that there may be a regulatory relationship between c-Jun and STC1 (Fig. 7E). The binding site of the transcription factor c-Jun was predicted in the promoter region of STC1 by the JASPAR database. Chromatin immunoprecipitation–qPCR in LM2 cells indicated that the enrichment efficiency of the transcription factor c-Jun in the predicted binding site of STC1 promoter region was much higher than that of IgG control, indicating that the transcription factor c-Jun can directly bind to the promoter region of STC1 (Fig. 7F). These results revealed that the activated JNK signaling pathway in lung metastatic breast cancer promotes the STC1 expression.

## Discussion

**Fig. 8:**
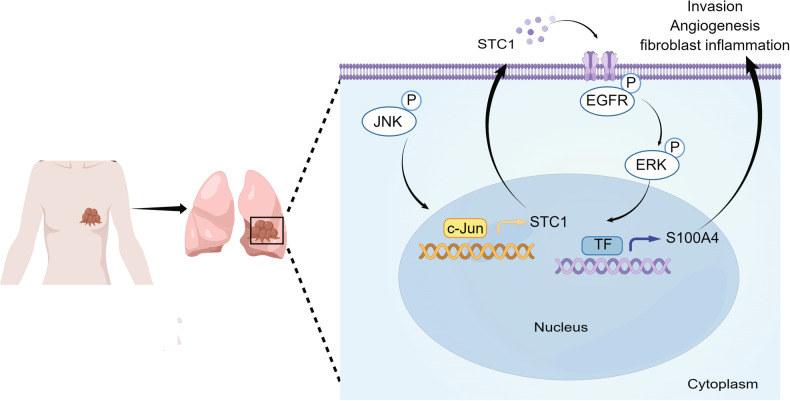
Schematic diagram depicting the roles of STC1 in breast cancer pulmonary metastasis.

Previous researches have recognized the involvement of STC1 in tumor progression. However, few studies have reported the role of tumor-derived secretory STC1 in tumor organ metastasis. In the present study, we report the role of tumor-secreted STC1 in pulmonary metastasis of breast cancer by enhancing the invasiveness of cancer cells and promoting angiogenesis and lung fibroblast inflammatory changes in the metastatic microenvironment. Breast cancer cells mediate the effect of STC1 on the tumor microenvironment. STC1 upregulates the expression of S100A4 in breast cancer cells by promoting EGFR phosphorylation and ERK signaling. S100A4 mediates the functions of STC1 on tumor cells and the metastatic microenvironment. We also found that in breast cancer cells with lung-tropism, activated JNK signaling upregulates STC1 expression (Fig. 8).

Pulmonary metastasis is a common organ metastasis of breast cancer [2], and TNBC is the subtype most prone to lung metastasis [3], so it is reasonable to use TNBC cell lines as a model for lung metastasis research. Moreover, TNBC is the most aggressive breast cancer subtype, notably, after tumor metastasis occurs, the clinical prognosis of patients is extremely poor, and there is still a lack of efficient targeted drugs for the therapy of TNBC in clinical practice [43]. Therefore, revealing the mechanism of pulmonary metastasis of TNBC is beneficial to provide new potential therapeutic targets for the treatment of breast cancer. Previous studies have indicated that higher STC1 expression in tumor tissues of breast cancer patients predicts poor recurrence-free and overall survival [44, 45]. This study found that the high expression of STC1 in tumor tissue was associated with poor LMFS in breast cancer patients, which has not been reported by previous works.

In the presented study, we found that STC1 promoted the aggressive properties of breast cancer cells in vitro and metastasis to the lungs in vivo, but did not affect breast cancer proliferation. Similar studies showing that STC1 did not affect tumor proliferation have been reported [39, 46]. However, there is also evidence indicating that STC1 can significantly promote breast tumor growth [27]. The reason for this inconsistency may be the difference in the animal models used. The tumor-bearing mice used in Chang’s study were BALB/c wild-type mice [27], and we used BALB/c immunodeficient mice. This difference in immune status may be the main reason for the difference in tumor growth in animals. Recent work also indicated that STC1 is a novel immune checkpoint that inhibits the phagocytosis of dying tumor cells by antigen-presenting cells and impairs the antigen-presenting capacity of antigen-presenting cells and the activation of T cells, ultimately contributing to tumor immune escape and tumor growth [33].

According to previous studies, STC1 can activate PI3K/AKT and JNK/c-Jun signaling pathways to enhance the invasiveness of breast cancer cells [39, 46]. However, the mechanism responsible for STC1 promotion of breast cancer metastasis to the lungs has not yet been examined. Considering that STC1 can act as a tumor-secreted protein, we investigated the impact of STC1 on the tumor microenvironment of breast cancer lung metastases. We found that STC1 could promote the accumulation of vascular endothelial cells in lung metastases of nude mice. However, tube formation assay in vitro did not show the pro-angiogenic effect of rhSTC1 treatment, nor in STC1-overexpressed HUVECs. It has been reported that STC1 may achieve its oncogenic function in an autocrine manner [47]. Our experiments supported this point. The phenomenon we observed is different from that of some previous studies that claimed that STC1 can directly promote angiogenesis. Law et al. believed that STC1 could increase eNOS, VEGF, and VEGFR2 expression levels and stimulate the VEGF signaling pathway in vascular endothelial cells, thereby promoting angiogenesis [48, 49].

In the tumor microenvironment, fibroblasts may transform into tumor-associated fibroblasts [14, 15]. STC1 protein can be secreted by tumor-associated fibroblasts stimulated by PDGF to facilitate the growth and metastasis of colorectal cancer cells [29]. Fibroblast-derived STC1 regulates tumor-associated macrophages and lung adenocarcinoma development [50]. STAT3 induces the secretion of STC1 from tumor-associated fibroblasts to foster breast cancer growth [51]. These studies suggest that fibroblasts play a role in tumor development and that STC1 can mediate fibroblast function. In contrast, the effect of tumor cell-derived STC1 on stromal cells has been rarely reported. We found a phenomenon similar to the effect of STC1 on angiogenesis, that is, the effect of STC1 on the migratory movement of fibroblasts might also be mediated by breast cancer cells. In the tumor microenvironment, fibroblasts can be “educated” to exhibit pro-inflammatory properties that promote tumor growth and angiogenesis [14, 15]. STC1 is involved in inflammatory responses [52]. We found a pro-inflammatory effect of STC1 on lung fibroblasts, and this effect might be mediated by breast cancer cells.

Relying on transcriptome sequencing screening, we found that the target of STC1 in breast cancer cells is S100A4. S100A4 is considered to be an important driver gene of tumor metastasis, which can not only promote the invasiveness of tumor cells, but can also regulate the tumor microenvironment [37, 38]. Therefore, the involvement of S100A4 may convincingly explain the multifunctional regulatory effect of STC1 on breast cancer cells themselves and the tumor microenvironment of breast cancer lung metastases. We examined the regulatory effect of STC1 on S100A4, and found a STC1–EGFR–ERK–S100A4 signaling axis in lung metastatic breast cancer cells. Previous studies have shown that S100A4 may be involved in EGFR signal transduction [53, 54]. Recombinant protein S100A4 and extracellular S100A4 also lead to the phosphorylation of ERK [55]. In our study, we observed that STC1 upregulates S100A4 by promoting the phosphorylation of EGFR and ERK. This seems to imply that EGFR, ERK, and S100A4 may be in a positive feedback loop under certain conditions. A previous study has also shown that STC1 can promote the phosphorylation of JNK in tumors [39], but in our study, the effect of STC1 on JNK phosphorylation was not observed, whereas the phosphorylation level of ERK was significantly increased. ERK signaling pathway activation is downstream of EGFR activation [40], and we observed that STC1 could facilitate the phosphorylation of EGFR by rhEGF, but STC1 itself did not affect the phosphorylation of EGFR. This may indicate that STC1 cannot play the role of EGF-like ligands, but may enhance the function of EGFR ligands, and the specific mechanism of action remains to be verified.

S100A4 has been previously recognized to play a crucial role in promoting lung metastasis of breast cancer [56]. We found that the functions of STC1 in breast cancer cells are mediated by S100A4. Our data indicated that STC1 acted on the tumor microenvironment indirectly, whereas S100A4 plays a clear role in regulating the tumor microenvironment [57]. So, S100A4 may also mediate the regulatory function of STC1 on the tumor microenvironment. S100A4 has the function of promoting angiogenesis [58], and our study indicated that S100A4 could mediate the pro-angiogenic function of breast cancer cells with high STC1 expression. S100A4 is also an important fibroblast activator [59]. Our findings showed that S100A4 can mediate the effect of breast cancer cells with high STC1 expression on lung fibroblasts. These indicated that S100A4 mediates the functions of STC1 in breast cancer cells and tumor microenvironments. Recent studies have shown that targeting S100A4 has potential application value in tumor treatment [60, 61], and the regulatory association between STC1 and S100A4 proposed in this study also provides theoretical support for targeting S100A4 for tumor treatment.

The lung is a relatively high-oxygen environment and encounters more external stimuli [41]. After metastasizing to the lungs, tumor cells must adapt to this environmental stress to survive and develop [62, 63]. Extracellular stimuli often lead to the activation of the MAPK signaling pathway [42]. We found that the MAPK signaling pathway was activated in lung metastatic breast cancer cells. STC1 is a cellular stress response factor [21]. Whether activated MAPK signaling in lung metastatic breast cancer cells is an initiating factor for STC1 upregulation is worthy of investigation. We found that STC1 is upregulated by the activated JNK signaling, which is upstream of STC1. This is different from a previous study that showed that STC1 can promote p-JNK and that the JNK signaling pathway is downstream of STC1 [39]. We speculated that MAPK signaling pathway activation might be an adaptive strategy for breast cancer cells to cope with environmental stress after metastasis to the lung, but the exact mechanism remains to be revealed.

In conclusion, our data establish the important role of STC1 in lung metastasis of breast cancer through its action on tumor cells and tumor microenvironments. Our study further demonstrates that tumor-secreted proteins are important participants in tumor metastasis.

## Supplementary information

Supplementary material

Figure S1

Figure S2

Original western blots

aj-checklist

## Data Availability

Data will be made available on request.
