# Supplementary material for: Stanniocalcin 1 promotes lung metastasis of breast cancer by enhancing EGFR–ERK–S100A4 signaling
Source: Cell Death Dis. 2023 Jul 4;14(7):395. doi: 10.1038/s41419-023-05911-z (PMC10318045; doi:10.1038/s41419-023-05911-z)
Supplement: Supplementary file 1 — Supplementary material [file 41419_2023_5911_MOESM1_ESM.doc]

**SUPPLEMENTARY MATERIALS**

**Stanniocalcin 1 promotes lung metastasis of breast cancer by enhancing EGFR–ERK–S100A4 signaling**

Anfei Liu^a, 1^, Yunting Li^a, 1^, Sitong Lu^a^, Chunqing Cai^a^, Fei Zou^a^, Xiaojing Meng ^a, *^

^a^ Department of Occupational Health and Occupational Medicine, Guangdong Provincial Key Laboratory of Tropical Disease Research, School of Public Health, Southern Medical University, Guangzhou, Guangdong510515, China

^1^ These authors contributed equally to this work.

* Corresponding authors：

Xiaojing Meng, Department of Occupational Health and Occupational Medicine, School of Public Health, Southern Medical University, 1838 Guangzhoudadaobei, Guangzhou, Guangdong, China. Tel: +8620-61648471; fax: +8620-61648324. Email: xiaojingmeng@smu.edu.cn.

**Figure S1. Effect of STC1 on proliferation of breast cancer cells.** (A) Cell viability and proliferation of MDA-MB-231 and LM2 treated with rhSTC1 were measured by CCK-8 assays. (B) Proliferation of MDA-MB-231 with STC1 overexpression and LM2 with STC1 downregulation, as measured by CCK-8 assays. (C)Volume and weight of tumors formed by STC1 overexpressed MDA-MB-231 cells in nude mice were measured. The p values were obtained by unpaired *t* test or one-way ANOVA analysis with Dunnett's multiple comparisons test, or repeated measures two-way ANOVA analysis. ns, no significance, p > 0.05.

**Figure S2. The pro-metastatic function of S100A4.** (A) Validation of S100A4 overexpression and knockdown in human breast cancer cell lines, MDA-MB-231 and LM2, by Western blot analyses. (B) Transwell invasion of S100A4-overexpressed MDA-MB-231 and S100A4-downregulated LM2 cells. (C) Tube formation of HUVECs co-cultured with S100A4-overexpressing MDA-MB-231 or S100A4-downregulated LM2. (D) Transwell migration of MRC5 co-cultured with S100A4-overexpressing MDA-MB-231 or S100A4-downregulated LM2. (E) The relative mRNAs expression of inflammatory factors IL1B, IL6, IL8 in MRC5 co-cultured with S100A4-overexpressing MDA-MB-231 or S100A4-downregulated LM2. The p values were obtained by unpaired t test. Scale bars, 100 μm. ** p < 0.01; *** p < 0.001.

**Supplemental Table 1. Sequences of mRNA primers.**

| Gene | Sequence |
| --- | --- |
| GAPDH | Forward: GTCTCCTCTGACTTCAACAGCG |
|  | Reverse: ACCACCCTGTTGCTGTAGCCAA |
| STC1 | Forward: GCAGGAAGAGTGCTACAGCAAG |
|  | Reverse: CATTCCAGCAGGCTTCGGACAA |
| IL1B | Forward: CCACAGACCTTCCAGGAGAATG |
|  | Reverse: GTGCAGTTCAGTGATCGTACAGG |
| IL6 | Forward: AGACAGCCACTCACCTCTTCAG |
|  | Reverse: TTCTGCCAGTGCCTCTTTGCTG |
| IL8 | Forward: GAGAGTGATTGAGAGTGGACCAC |
|  | Reverse: CACAACCCTCTGCACCCAGTTT |
| VEGFA | Forward: TTGCCTTGCTGCTCTACCTCCA |
|  | Reverse: GATGGCAGTAGCTGCGCTGATA |
| S100A4 | Forward: CAGAACTAAAGGAGCTGCTGACC |
|  | Reverse: CTTGGAAGTCCACCTCGTTGTC |

**Supplemental Table 2. The antibodies used for western blot or IHC.**

| Protein | Company | Catalog Number | Application |
| --- | --- | --- | --- |
| GAPDH | Proteintech | 60004-1-Ig | WB |
| STC1 | Proteintech | 20621-1-AP | WB, IHC |
| S100A4 | Proteintech | 66489-1-Ig | WB, IHC |
| p-JNK | Cell Signaling | 4668 | WB, IHC |
| JNK | Cell Signaling | 9252 | WB |
| p-ERK | Cell Signaling | 4695 | WB, IHC |
| ERK | Cell Signaling | 9102 | WB |
| p-p38 | Cell Signaling | 4511 | WB |
| p38 | Cell Signaling | 8690 | WB |
| p-EGFR | Cell Signaling | 3777 | WB |
| EGFR | Cell Signaling | 4267 | WB |
| α-SMA | Abcam | ab124964 | IHC |
| CD31 | Abcam | ab182981 | IHC |
| LY6G | Abcam | ab238132 | IHC |
