# Supplementary figures and images for: Stanniocalcin 1 promotes lung metastasis of breast cancer by enhancing EGFR–ERK–S100A4 signaling
Source: Cell Death Dis. 2023 Jul 4;14(7):395. doi: 10.1038/s41419-023-05911-z (PMC10318045; doi:10.1038/s41419-023-05911-z)

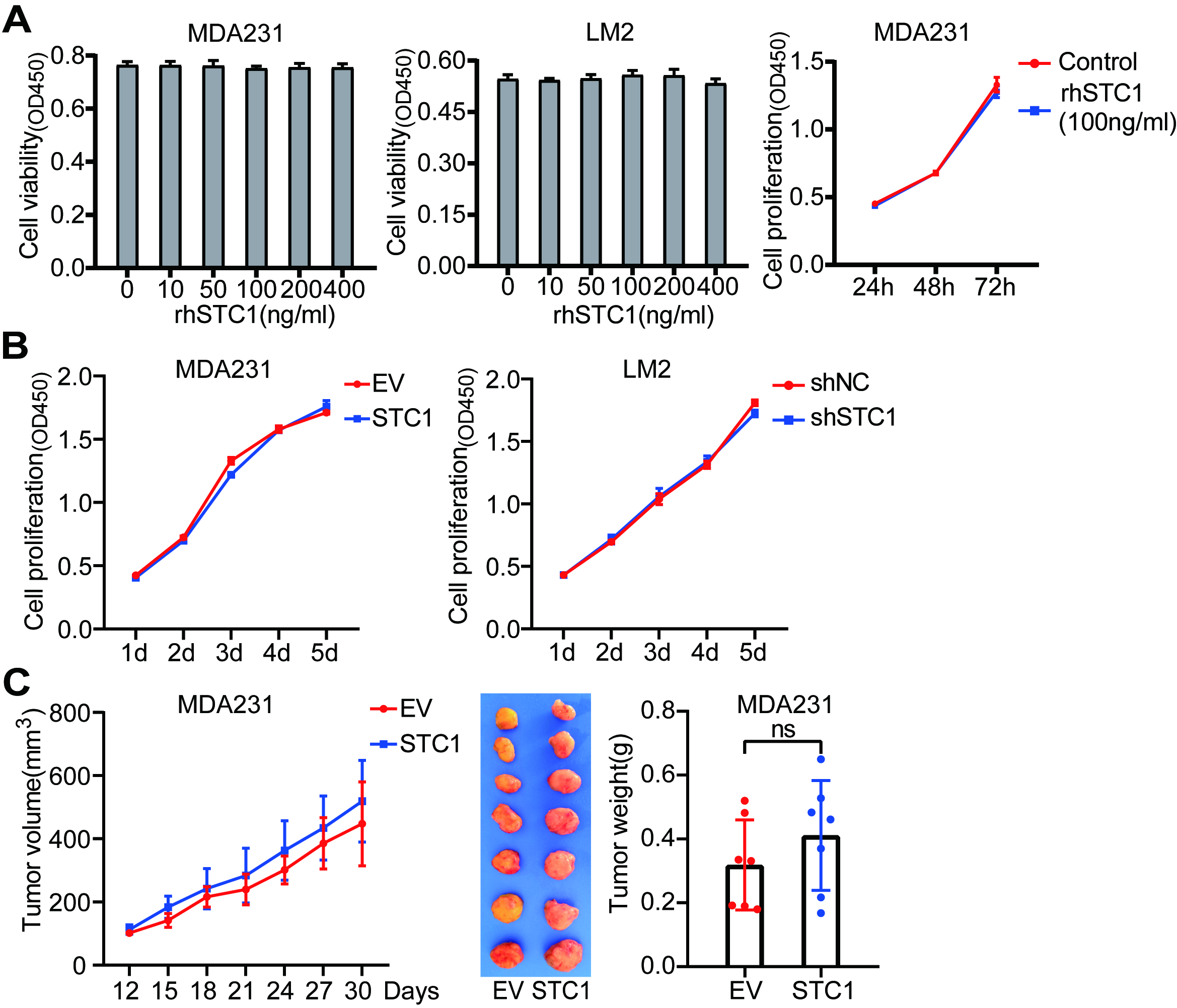

Supplement: Supplementary file 2 — Figure S1 [file 41419_2023_5911_MOESM2_ESM.tif]

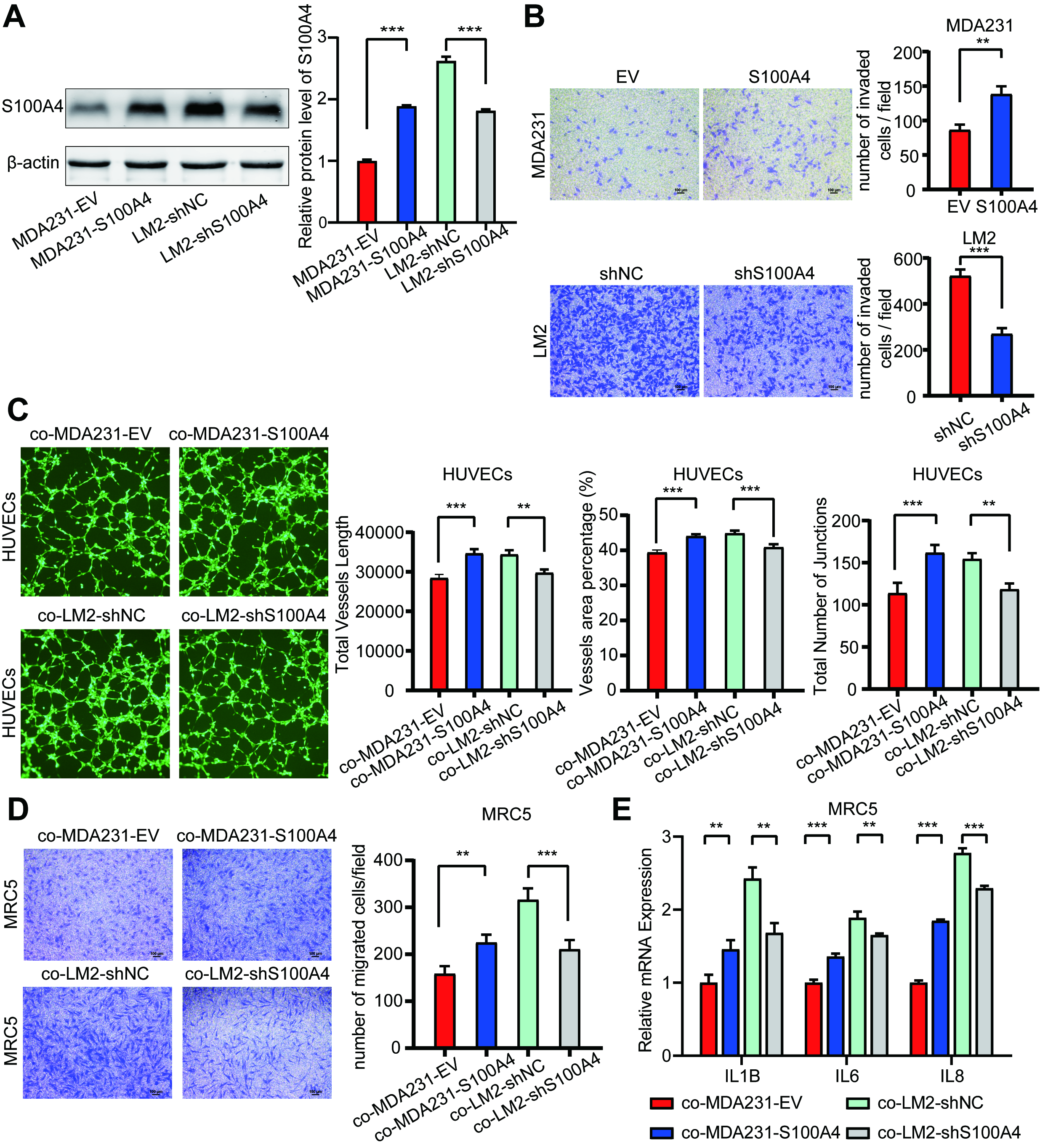

Supplement: Supplementary file 3 — Figure S2 [file 41419_2023_5911_MOESM3_ESM.tif]

Fig.1B

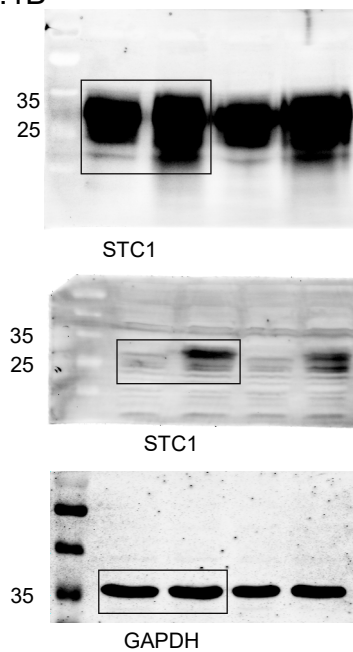

Fig.2B

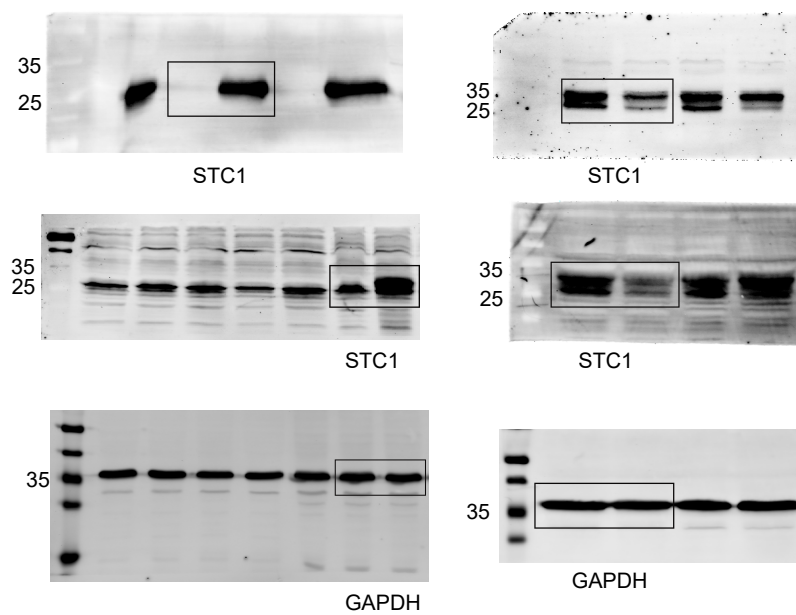

Fig.5E

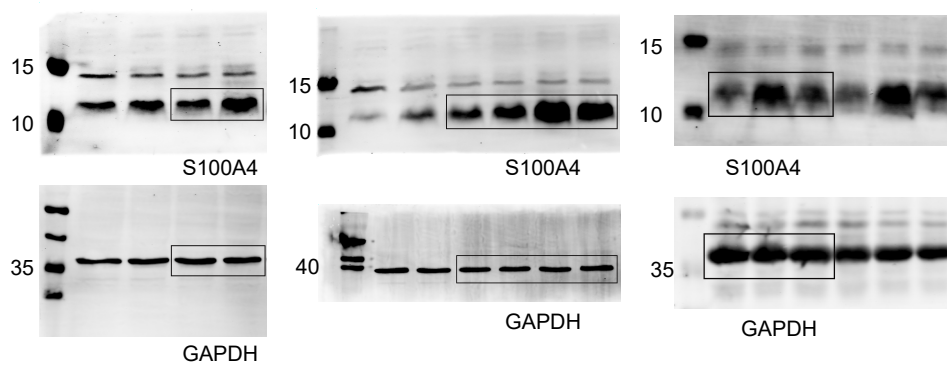

Fig.5G

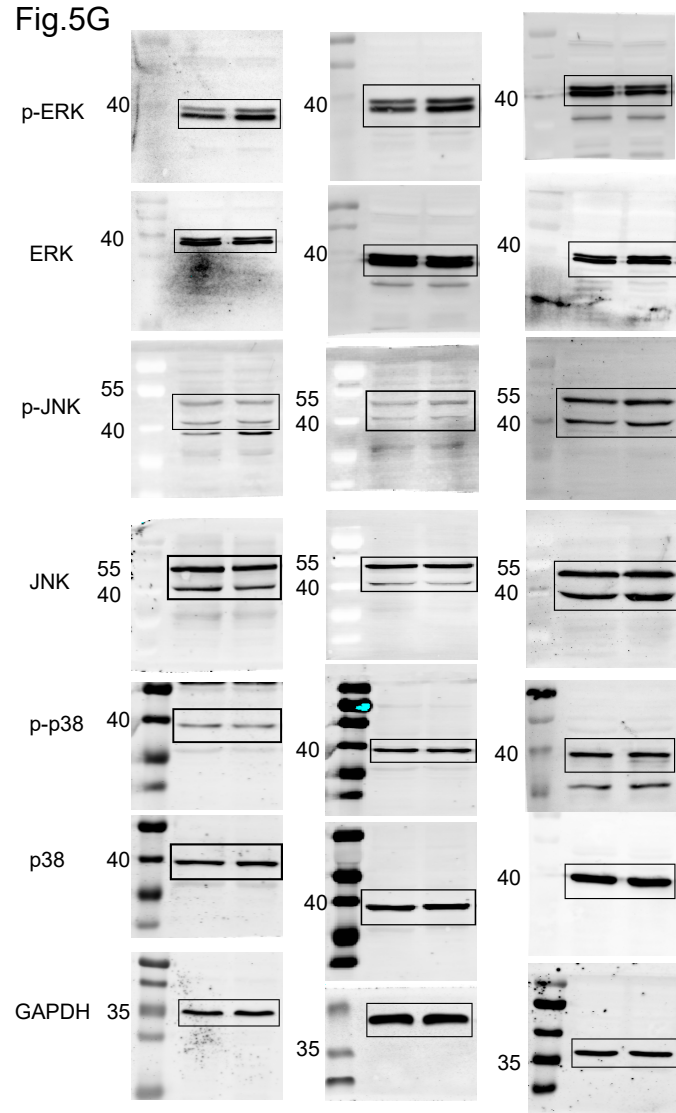

Fig.5H

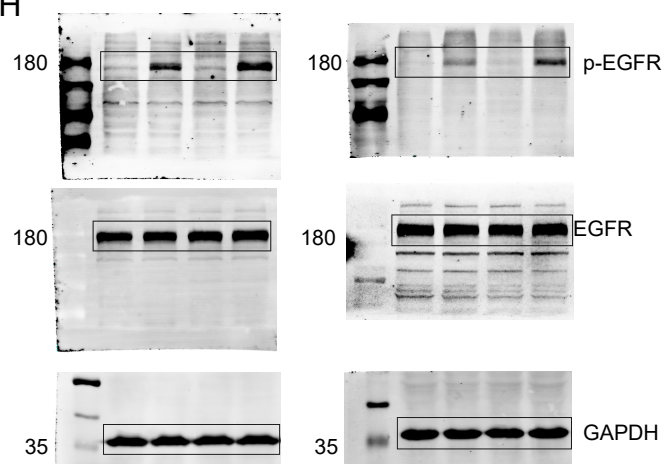

Fig.5I

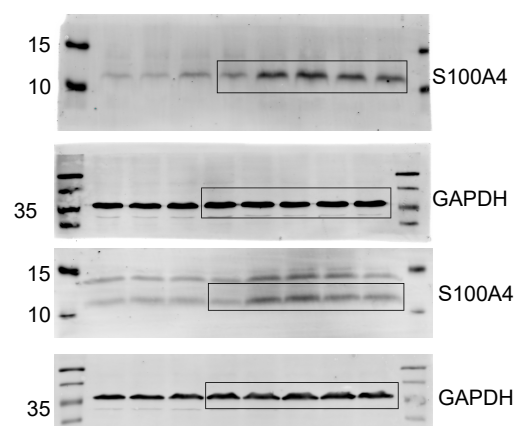

Fig.6A

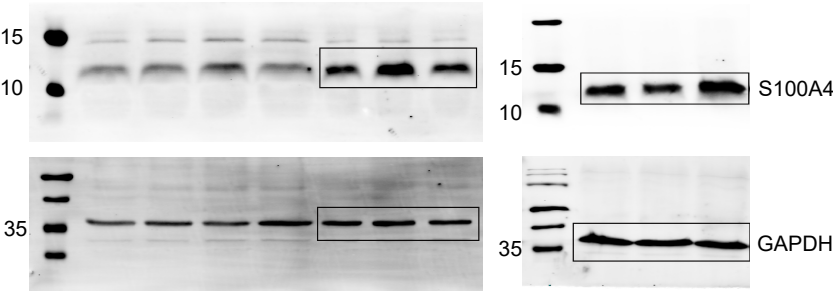

Fig.7A

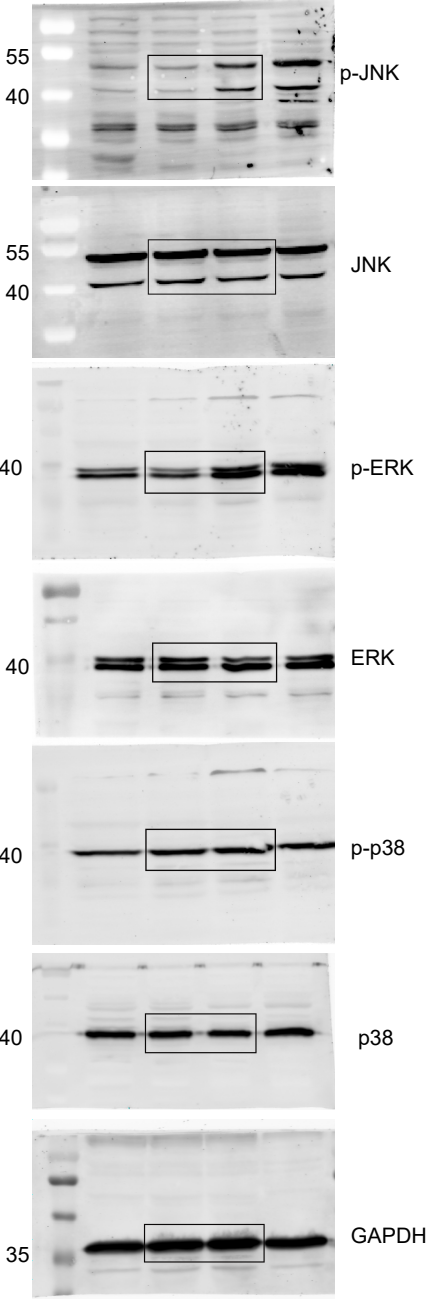

Fig.7C

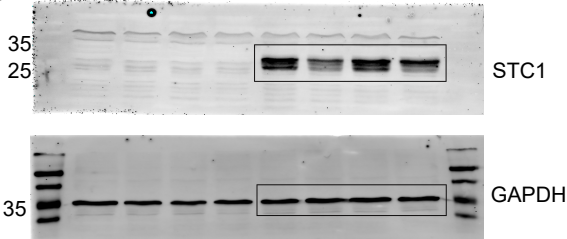

Fig.S2A

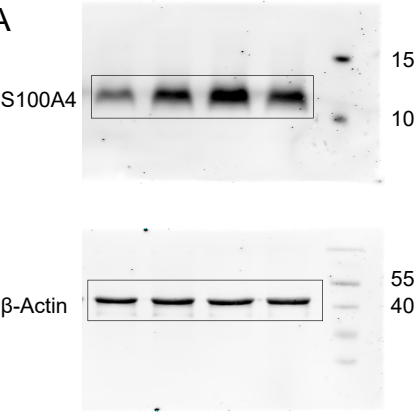

Supplement: Supplementary file 4 — Original western blots [file 41419_2023_5911_MOESM4_ESM.pdf]
